# Cancer patients’ return-to-work adaptation experience and coping resources: a grounded theory study

**DOI:** 10.1186/s12912-023-01219-7

**Published:** 2023-03-10

**Authors:** Jiashuo Xu, Yuwen Zhou, Jiamei Li, Jue Tang, Xiaoyun Hu, Yifan Chen, Yujie Guo

**Affiliations:** 1 Medical College (school of nursing), Nantong University, Nantong, Jiangsu China; 2 Department of Respiratory and Critical Care Medicine, Affiliated Hospital of Xuzhou Medical University, Nanjing, Jiangsu China; 3 Nursing Department of Taizhou Second People’s Hospital, Taizhou, Jiangsu China; 4 Pharmacy College, Nanjing Medical University, Nanjing, Jiangsu China

**Keywords:** Cancer, Return-to-work, Adaptation, Coping resources, Grounded theory

## Abstract

**Objective:**

To explore the return-to-work adaptation experience and coping resources used by cancer patients.

**Methods:**

With the help of the Nantong Cancer Friends Association, from June 2019 to January 2020, this study recruited 30 cancer patients who had returned to work using purpose sampling, snowball sampling and theoretical sampling. The researchers analyzed the data using initial-, focusing-, and theoretical coding.

**Results:**

The adaptation of cancer patients to return-to-work is a rebuilding process by taking advantage of the available personal and external coping resources. The adaptation experience includes: focusing on rehabilitation, rebuilding self-efficacy, and adjusting plans.

**Conclusion:**

Medical staff should help patients mobilize coping resources to adapt to return to work.

## Background

Cancer is a global public health problem of significance [1]. A trend of increasing incidence of cancer in the younger population has been observed [2]. Owing to rapid economic development, improved livelihood, and shifts in risk factors, the 5-year survival rate for cancer has dramatically increased in recent decades of China [3]. As cancer survival rates increase, the reduced working life span of patients due to the disease should be taken into account, one studies have shown that cancer patients have a 1.42-fold greater risk of unemployment than the general population [4]. Unemployment will make cancer patients feel socially isolated [5], reduce quality of life [6] and increase the economic burden on individuals and society [7]. Thus, it is important to explore the problems faced by cancer patients when they try to return to work after the treatment and their adaptation strategies and coping resources.

The more familiar concept of returning to work has been described to consist of four key phases: off work, re-entry, maintenance, and advancement [8]. Return to work is regarded as a mark of complete recovery as the patients are able to regain their social lives, carry out pre-illness daily activities and specific social tasks [9]. Return-to-work can improve cancer patients’ quality of life and subjective physical and mental health [10]. It can also reduce employers’ friction costs and enhance society’s overall productivity [11]. The ability to adapt to new situations is an important indicator of cancer patients’ health status and quality of life and plays a vital role in successful psychosocial rehabilitation of cancer patients [12]. There have been qualitative studies exploring the specific stresses that cancer patients experience in adapt to returning to work [13]. For cancer patients, the ability to adapt to new circumstances, take advantage of emerging opportunities, and finding meaningful occupational activities was crucial [14]. However, changes in their state of adaptation and the specific resources they use in the return-to-work process have not been studied further. Successful adaptation to return-to-work relies on comprehensive multidisciplinary resources [15]. This study explores cancer patients’ return-to-work adaptation experiences and coping resources used by them, and the results can serve as a theoretical basis for developing a framework for professional intervention.

## Method

### Research subjects

All procedures performed in studies involving human participants were in accordance with the ethical standards of the institutional and/or national research committee and with the 1964 Helsinki Declaration and its later amendments or comparable ethical standards. The study was approved by the Human Research Ethics Committee of the Affiliated Hospital of Nantong University (No.201,915). All participants provided written informed consent before participating in the study. Inclusion criteria for enrollment of patients in the study were: [1] age 18 to 60 years old; [2] diagnosis of cancer; [3] completed the treatment plan formulated by the physician based on the condition, and the results of the tumor imaging and/or markers showed that only regular monitoring is required; [4] working before treatment and had returned to work by the start of the study; [5] understand their own conditions; The exclusion criteria were: [1] other severe systemic complications and [2] diagnosis of stage IV cancer. The sample size is based on data saturation. From June 2019 to January 2020, with the support of the Nantong Cancer Friends Association, the researchers visited cancer patients’ group exercise sites (where patients usually work out in pairs) and the Cancer Friends Association’s science halls (where patients usually share their recovery experiences with each other) in the field. Then, using purposive sampling, the researcher recruited the first three patients who were considered to be informative. After completing interviews with these three patients, the researcher used snowball sampling to obtain contact information for additional patients and to schedule appointments with them for interviews. Data saturation was achieved when interviews with 25 patients were finished. To build the theory and refine it, the researchers recruited 5 patients with some special characteristics and experiences by theoretical sampling. Finally, 30 patients (numbered N1 to N30 according to the interview order) participated in the study. (Table 1).

**Table 1:** Basic information of interviewees

Serial number	Age	Gender	Cancer site	Cancer staging	Treatment mode	Work before illness	Current Job	Time to end treatment (months)
N1	56	Woman	Breast	III	R、C、T	Teacher	Teacher	119
N2	42	Man	Lung	III	R、C、T	Civil servants	Civil servants	26
N3	49	Woman	Breast	II	Op、C、R	Rear-service	Librarian	40
N4	46	Woman	Thyroid	III	Op	Worker	Rear-service	190
N5	50	Woman	Ovary	III	Op、C	Civil servants	Civil servants	41
N6	53	Man	Nasopharynx	II	Op、C、R	Self-employed	Engineering contractor	25
N7	58	Man	Lung	III	Op、C	Cook	Retailer	61
N8	53	Man	Rectum	III	Op、C、R	Retailer	Retailer	74
N9	59	Man	Rectum	II	Op、C	Civil servants	Civil servants	59
N10	47	Woman	Breast	III	Op、C、R	Civil servants	Civil servants	63
N11	57	Man	Colon	II	Op、C	Worker	Cemetery keeper	97
N12	34	Woman	Breast	II	Op、C	Company staff	Company staff	43
N13	34	Woman	Breast	III	Op、C	Civil servants	Civil servants	49
N14	43	Woman	Breast	I	Op、C	Company staff	Insurance salesman	72
N15	55	Man	Liver	III	C、Op	Street clerk	Street clerk	35
N16	59	Man	Stomach	II	Op、C	Teacher	Volunteer	61
N17	53	Woman	Breast	III	Op、C、R	Enterprise leaders	Enterprise leaders	48
N18	49	Woman	Lung	III	Op、C、R	Self-employed	Self-employed	73
N19	50	Woman	Cervix	II	Op	ICU nurse	Orthopedic nurse	84
N20	41	Woman	Skin	II	Op	Nurse	Nurse	181
N21	27	Woman	Gullet	II	Op	Doctor	Doctor	61
N22	40	Woman	Breast	II	R、E、Op	Head nurse	Nurse	60
N23	35	Man	Rectum	III	Op、C、R	Doctor	Doctor	72
N24	36	Woman	Stomach	III	Op	Doctor	Researcher	36
N25	52	Woman	Colon	III	Op、C	Doctor	Researcher	34
N26	46	Man	Thyroid	II	Op	Teacher	Teacher	33
N27	45	Woman	Cartilage	II	Op、C	Teacher	Teacher	71
N28	40	Man	Gullet	I	Op	Teacher	Teacher	47
N29	49	Woman	Rectum	III	Op、C	Teacher	Teacher	123
N30	55	Man	Lung	II	Op、C、R	Enterprise leaders	Enterprise leaders	95

注: Note: Op: operation, C: chemotherapy, R: radiotherapy, T: targeted therapy, E: endocrine therapy

### Researcher role

The researcher needs to reflect on his or her role as a research instrument in qualitative research [1]. The role of the researcher, the person present when the interviews were conducted in this study was a nursing master’s student who had completed training and obtained a certificate in qualitative research [2]. Relationship with the study participants. The researcher established a closer relationship with the patients through the preliminary field visits and online contact, and because of the study and internship experience, the researcher was seen by the patients as a more professional medical practitioner [3]. The perceptions of cancer patients’ return to work were mainly derived from clinical internship experience during undergraduate studies and relevant literature reading during graduate studies. During the conduct of the study, the researcher also avoided subjective bias and increased sensitivity to the research questions by keeping a reflective journal, memos, and participating in group discussions.

### Data collection

The constructivist grounded theory was used to collect and analyze data, it is a bottom-up approach to construct a theory that allows for close integration of theoretical research and practical aspects of return-to-work with cancer patients. The study data were collected by face-to-face interviews. Most of the interview locations were in cafes, quiet corners of parks, patients’ offices, etc. The interview environment meets the conditions of quietness, privacy, and not easily disturbed, which is conducive to recording. Each interview lasted for about 30 min. Before the interview, the researcher spent approximately five minutes presenting the purpose of the study and creating a relaxed atmosphere. The outline of the interviews was determined through literature review [16] and pre-interviews. Researchers began interviews with preliminary questions such as *“How are you recovering now? "* And *“How do you feel about returning to the workplace after cancer?“*. The main questions were then posed: *“How are you adapting to your return to work after having cancer?“, “What resources are available to help you return to work?“* In interviews, the researchers responded positively to patient responses, but did not interrupt or comment. The patients’ facial expressions and body language were observed and questions were constantly updated during the interview according to the information provided by the interviewee.

### Data analysis

After each interview, researchers transcribed the recordings word by word. A total of 119,980 words were transcribed in the study. Interviewees’ tone, pauses, and other relevant information was also used to assist the analysis. MAXQDA software was used to analyze the data. First set of codes were generated by assigning code to each line of an interview (line-by-line coding). The most frequent and critical codes were further classified and integrated into focus codes. The theoretical coding was achieved by combining theoretical sampling and memo information. At the theoretical coding stage, charts were used to express results to analyze deficiencies. After deficiencies were found, the researchers use continuous comparisons between data and results to improve the accuracy of the analysis.

## Results

**Fig. 1:**
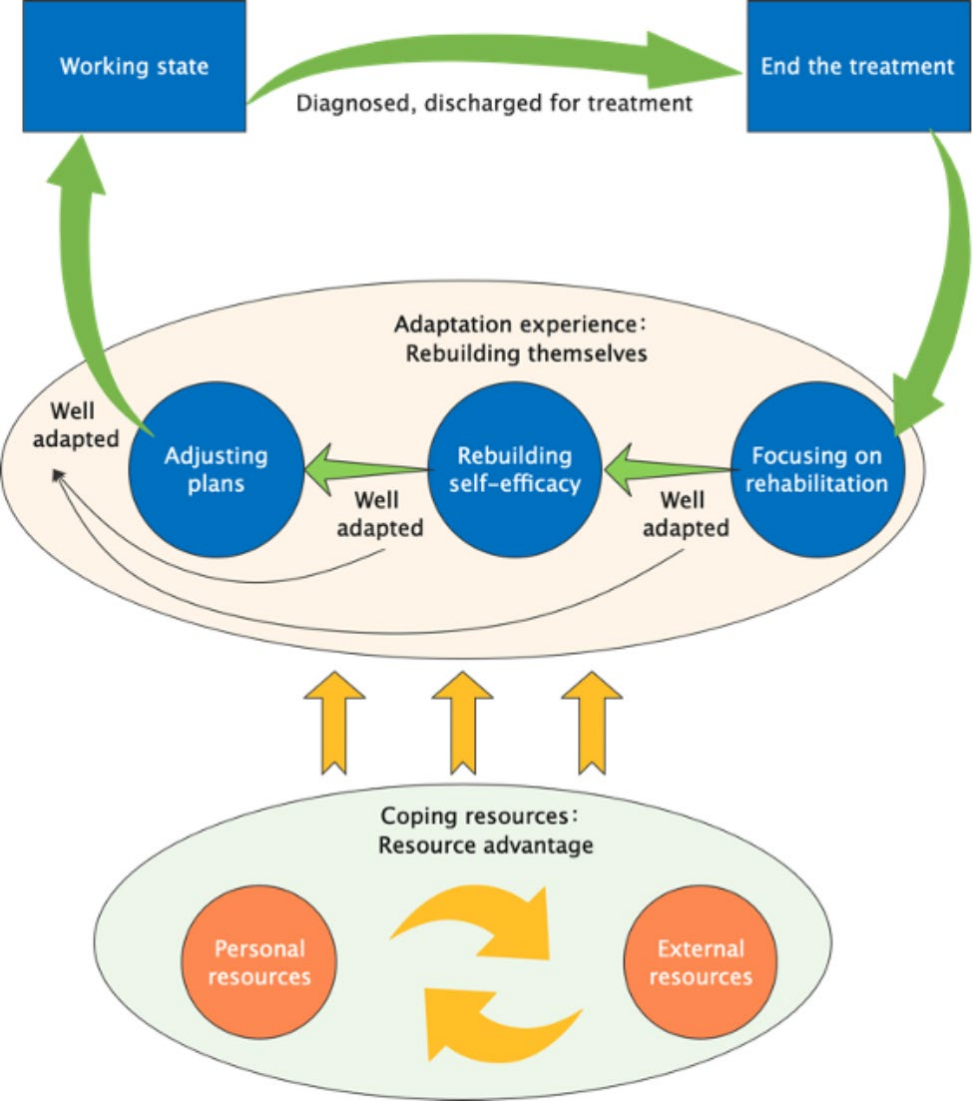
Cancer patients’ return-to-work adaptation experience and coping resources

After data analysis, the relationship between the categories was gradually clarified (Fig. 1). After the end of treatment, the patients prioritized rehabilitation. Their self-efficacy had decreased due to cancer trauma, so they tried to rebuild their self-efficacy for return-to-work. Due to the shadow of cancer, it is difficult for patients to return to work without changes to their previous lifestyles. They adjust their lifestyles to maintain a balance between health and career. The patients use coping resources throughout the process of return-to-work adaptation. Personal resources provide a continuous internal driving force, and external resources provide social support. The adaptation of cancer patients to return to work is mainly a process of rebuilding themselves by making the most of the available resources.

### Adaptation experience

#### Focusing on rehabilitation

Cancer and its treatment is a major physical and psychological trauma to patients. Immediately after the treatment, patients need to focus on rehabilitation, which is their starting point for life after cancer treatment and runs through the entire process of returning to work. It includes “healing”, “introspection and change”, and “strengthening learning”, which influence each other and act as cause and effect to each other. After the treatment was completed, the patients discovered changes in their physical functions. In interviews, the patients often used expressions like “I used to be…, but now….“ Patients were eager to recover their health, so they introspected on past unhealthy behaviors. The insights gained through introspection motivate the patients to strengthen their learning and acquire positive behaviors. Under a joint action of these three aspects patients gradually recover their physical and mental health.

1. Healing

Cancer patients experience a series of side effects during and after the treatment. Vomiting, hair loss, insomnia, and fatigue are very common in patients undergoing therapy. Cancer treatment kills cancer cells, but at the same time, destroys the healthy cells, causing weakness. “Easily tired”, “unable to stand straight”, and “no strength to speak” were the frequent expressions of physical weakness. The focus of this stage is to overcome the feeling of weakness through diet and exercise, which is required for adapting to return to work.

*“After completing the treatment, I was fragile and I felt like stepping on cotton while walking. It took more than two months to get better” (N1)*.

*“I developed radiation pneumonia, my resistance was feeble, and I coughed a lot. There was no way to treat it, so I strengthened my nutrition, exercised, and rested adequately.“ (N2)*.

*“After the operation, I often couldn’t fall asleep in the night due to acid reflux. I felt comfortable only in the sitting position. I didn’t rest well at night, and I was sluggish during the day. Later, the symptoms were relieved through dietary conditioning.“ (N16)*.

(2) Introspection and change

Patients reflect on the factors that led to their illness and take steps to eliminate them. Patients make changes to work and rest schedules, nutrition, emotional life, and exercise.

*“In the past, it was common for me to stay up late to work overtime. After I got sick, I understood the importance of regular work and rest. I retire at around 11 o’clock now.“ (N9)*.

*“I used to be an inactive person, but now I run for an hour every day, rain or shine.“ (N23)*.

*“In the past, I was concerned about recognition from others. If tone of others towards me was unfriendly, I wondered if I had done something wrong. I now understand that I should live for myself, and my health and happiness are most important now.“ (N25)*.

(3) Strengthening learning

Patients have a strong desire to acquire knowledge related to rehabilitation. Patients improve cognition through learning, which promotes healthy behaviors. Physical discomfort and emotional distress motivate patients to strengthen their understanding of rehabilitation. Patients ensure that their strategies for dealing with stress are up to date through learning. It was found that patients wanted to learn about rehabilitation to promote health, psychological understanding to manage destructive emotions, and traditional Chinese culture to maintain a good attitude in life.

*“Because I am a medical graduate, I have a stronger sense of learning than ordinary patients. When I felt uncomfortable about something, I consulted my graduate supervisor, and read the relevant literature.“ (N25)*.

*“Now there are many psychological counseling courses for cancer patients on the Internet that teach ways to manage emotions. I think these courses are useful and I recommend them to other patients.“ (N3)*.

*“After studying Tao Te Ching, I slowly learnt to look down on unimportant things. Maintaining a good attitude every day, and being happy is most important now.“(N12)*.

#### Rebuilding self-efficacy

Self-efficacy is a subjective judgment of whether an individual is competent for specific behavior. After treatment, patients’ self-efficacy of return-to-work was significantly reduced. Rebuilding this self-efficacy which includes ‘learning from other patients’, ‘feeling emotional support’, and ‘confidence training’, is key to adapting to return-to-work.

1. Learning from other patients

Patients reported the influence of other cancer patients on them. They were willing to get along with those who have returned to work after cancer treatment to gain indirect experience and improve confidence. When patients are apprehensive of returning to work, the influence of role models is more significant. Patients integrate the experience gained from the role models to guide behavior and improve the confidence of returning to work.

*“My mind becomes more positive with patients who have returned to work. I feel hopeful in life and feel confident in going back to work.“ (N16)*.

*“We worry that fatigue is detrimental to recovery. But seeing their mental outlook improve, some of our concerns are assuaged.“ (N28)*.

(2) Feeling emotional support

Emotions are an essential means of interpersonal communication. Emotional support from others play an important role in inculcating a positive intention to return to work. It motivates the patients to realize their self-worth by returning to work and increases their confidence. *“I want to thank my family for taking good care of me after I fell ill. They encouraged me to go back to work, and my parents moved in to support me. It made me feel important and needed.“ (N6)*.

*“My doctor cared about my recovery. He told me often that he hoped I could return to a normal life.“ (N15)*.

*“The friendship between patients is precious. We encourage and comfort each other, just like holding each other’s hand and walking in the dark together. Once I was particularly anxious, a patient took the initiative to call me and encouraged me go back to work and live a full life. We talked on the phone for four hours, which moved me very much.“ (N23)*.

(3) Confidence training

Confidence training reinforces confidence, reduces passive and anxious responses, and enables one to respond positively to a specific situation. Cancer patients desire to increase income and realize self-worth, but they worry that their bodies would not adapt to work demands and that cancer will return due to work stress. This state of conflict and anxiety can be reduced by confidence training.

*“During sick leave, my colleagues used to come to consult me about work problems. I could solve the problems easily that they found difficult. This helped me gradually regain my confidence.“ (N12)*.

*“I worried that I would be late to work or leave early for health reasons, which would burden my work unit. So I worked from home, as per work hours to let my body adapt. It was strange at first, but I was happy once I got used to it.“ (N28)*.

#### Adjusting plans

It is difficult for patients to return to their original work without adjustments in other areas of life. Patients adjust plans to maintain a balance between health and career. Adjusting plans makes return-to-work easier and more feasible. It includes adaptation experiences such as ‘seeking support’ and ‘step by step approach’. Seeking support refers to outside help, which can provide a healthy working environment. Step by step approach refers to the patients’ self-regulation, which can reduce work pressure. As a result of these two factors, patients adapt to return to work and remain stable.

1. Seeking support

Patients seek support to reduce problems they may encounter at work.

*“You can look at my desk, and this is what I need to sort out by the day after tomorrow. It’s a lot less work than it was before I got sick. I expressed my difficulties to my boss and hoped to be transferred to a less demanding position. The boss said there was no suitable position, but he relieved a lot of my workload, for which I am very grateful.“ (N13)*.

*“It’s unrealistic for me to work like others. I discussed with my family to invest in me to run a clothing store. My family gave me a great support. I sit in the store selling clothes every day, which is very easy.“ (N18)*.

*“It’s not easy for me to find a job at my age, so I asked a friend to help me find a suitable job. With his help, I am now teaching Taijiquan to cancer patients here.“ (N16)*.

(2) Step by step approach

When asked about career plans, the patients said they do not have career plans. They will decide about the career based on their physical recovery in the future. Step by step approach embodies what Lao Tzu said, let nature take its course.

*“I have regrets. Before I got sick, I had a senior vice title for many years. At that time, I wanted to have a senior title before I retired. But now I have given up that goal, due to the pressure that comes with it, such as having to keep doing research and writing articles. I put my health first now.“ (N1)*.

*“According to the scale of the business before my illness, it was no problem for me to be the general agent. Yet, I scaled-down my business to half, which is tantamount to giving up the goal I set for myself. Now I just want to have good health. It will still be not too late to expand the business when my health is better.“ (N8)*.

### Coping resources

#### Personal resources

1. Cognition

Cognition affects the coping behavior and emotional attitude of patients adapting to return-to-work. In the first stage, patients improve their cognition by strengthening learning to deal with rehabilitation challenges. In the second stage, patients’ self-efficacy increases with further cognitive improvement. In the last stage, the patient’s cognition develops sufficiently to maintain health and work balance.

*“Participating in this psychological counseling class is useful. I will no longer be immersed in negative emotions as easily as before.“(N3)*.

*“I was much entangled at first. After listening to the doctor’s advice, I think I should return to work.“(N19)*.

*“If tired, I will immediately reflect if I have been too busy at work recently, and remind myself to take more rest. I won’t ignore it as I did before.“(N20)*.

(2) Faith

Faith is an individual’s firm belief in something. It fosters a sense of responsibility and rekindles patients’ desire to restore the social function. Faith can motivate the patient to inculcate a positive attitude and show behavioral confidence. Faith influences motivation to return to work.

*“Once, my son said to me, ‘Mom, I’ll study hard in the future, so as not to make you angry.‘ after he went out, I buried my head in my pillow and cried. I swear that I will work hard to earn money for him to grow up.” (N24)*.

*“In my heart, I always firmly believe that a leader like me must be responsible for his subordinates. The employees have supported me for so many years. I must go back to work and never want to give up.“ (N30)*.

(3) Resilience

Resilience enables individuals to recover and cope with severe stress and danger. Patients actively responded to various challenges in returning to work, adjusting themselves, restoring biological-psychological-mental homeostasis, and finally successfully returning to work.

*“I am a person who has died once. Anything that can’t beat me will make me stronger. I’m not afraid of death. What else can beat me?“ (N4)*.

*“When I encounter any problems, I actively find ways to solve them. As long as I think I am a healthy person, there is no reason for others to treat me as a patient.“ (N22)*.

(4) Belief

Belief refers to religious belief in this study. Positive religious belief can help in developing an effective coping style. In this study, three patients believed in Buddhism. They used the Buddhist perspective to view adverse events, generating positive experiences like reducing mental pain, improving sleep quality, and promoting interpersonal communication.

*“The Buddha said that bad fate can be changed by doing more good things, which gave me spiritual comfort.“ (N11)*.

*“If I am upset because of conflicts with others at work, I will recall Buddhist scriptures to calm my mind.“ (N22)*.

#### External Resources

1. Family support

Cancer patients’ loss of income results in a significant financial burden on their families. Families care for the patients during their illness and provide financial, emotional, and operational support which is evident at every stage of their return to work. When the patient returns to work, the family also returns to a state of balance and stability.

*“Cancer is not a struggle of the person who is suffering, but the whole family is fighting. Without the tolerance of my other half, my family may break up. So if we are able, we must work again to earn money to support our family.“ (N7)*.

*“The person I am most grateful for is my husband. After the radiotherapy my body suffered from edema. My husband helped me with wet compresses every day and accompanied me to the park to sunbathe. We go out for a walk and chat every day after dinner. Since I got cancer, I can understand the value of mutual support between husband and wife better.“ (N17)*.

(2) Professional support

Patients seek professional support from medical staff for clarifying their doubts about recovery, treatment, rehabilitation, and return to work. Professional support enables the patients to have access to correct information regarding their health. Patients also look up to the professional staff for emotional support. The patients reported that medical staff evaluated their recovery, made specific suggestions for improving health, and encouraged them to return to work. This helped the patient to make positive decisions about returning to work.

*“The doctor constantly told me what to pay attention to when I recovered. The last time, the doctor said I am recovering well and could consider going back to work.“ (N4)*.

*“I always worried that returning to work will affect my recovery. The doctor gave me the right information and guided me correctly about my condition. I felt like I had taken a reassurance pill, and I felt more relieved.“ (N15)*.

(3) Workplace support

Workplace support is an essential factor determining cancer patients’ return to work. Whether a patient can get this support or not is affected by the nature of the workplace. The care from superiors and colleagues reduced the work pressure and provided a warm working environment. Workplace support helped the patients to remain comfortable on their return to work.

*“My leader cared about me very much and let me have a good rest at home after leaving the hospital. During the sick leave, he and several colleagues visited me at home. He didn’t ask me to return to work immediately after the sick leave was over.“ (N29)*.

*“The leaders take good care of me and rarely schedule night shifts for me. My colleagues are also very considerate of me and don’t let me do heavy work.“ (N22)*.

(4) Peer support

Peers refer to people with the same living environment and experience who support each other for common goals. Many concerns about patients’ return-to-work may be difficult to understand by the average person, but peers can understand them. Peer support can make up for the shortcomings of other forms of social support. The friendly relationship between patients can promote their participation in social interactions. Peers can help others in their group with poor rehabilitation outcomes to implement self-management strategies. Patients who have successfully returned to work can encourage others to improve their self-efficacy.

*“I once thought I won’t be able go back to work. I had decided to take rehabilitation for the rest of my life, but my peers patiently persuaded me to have confidence in myself.“ (N7)*.

*“I didn’t want to go out before. I felt that I was sick and others would look at me differently, but now I look forward to meeting my peers every day.“ (N8)*.

## Discussion

The adaptation of cancer patients to return to work is a process of self-reconstruction by using their resource advantages. The core category of the adaptation experience of return-to-work for cancer patients is to rebuild themselves, and the specific categories are: focusing on rehabilitation, rebuilding self-efficacy and adjusting plans. Focusing on rehabilitation is the starting point for adapting to return-to-work, including: healing, introspection and change, and strengthening learning; rebuilding self-efficacy is the key for adapting to return-to-work, including: learning from other patients, feeling emotional support and confidence training; Adjusting plan is the guarantee for adapting to return-to-work, including: seeking support, step by step approach. The core category of coping resources for cancer patients returning to work is resource advantage, and the specific categories are: personal resources and external resources. Personal resources are coping resources possessed by the patients themselves, including cognition, faith, resilience and belief; external resources are social support resources that can be mobilized by patients, including: family support, professional support, workplace support and peer support.

### The significance of exploring the return-to-work adaptation experience and coping resources

The experience of cancer patients can best be explained as health–illness-transitions [17]. Self-management can influence a patient’s ability to respond to transitions in physical and emotional symptoms, spiritual well being, interpersonal relationships, and functional ability and lifestyle [18]. Previous studies have shown that self-management intervention can help patients with chronic pain return to work, and formulating a reasonable self-management plan is the key [19, 20]. The change of state model [21] proposes that each individual who strives to make behavioral change has different needs and motives. Therefore, this study explores the return-to-work adaptation experience and coping resources, which may help cancer patients develop reasonable self-management plans. According to the ‘adaptive leadership’ framework, medical staff at all levels help patients adapt to difficult situations by assuming leadership roles [22]. The results of this study can help the medical staff and other professionals involved in the care of cancer patients to assist them in adapting to return to work.

### Understanding the return-to-work adaptation experience of cancer patients

#### Focusing on rehabilitation is the starting point for cancer patients to return to work

The patient’s physical and mental health after treatment affects the outcome of adaptation. A healthy lifestyle is the key to controlling cancer progression [23]. During rehabilitation, the medical staff generally focuses more on the physical aspects rather than following a more balanced approach that includes both physical and mental aspects. The patient’s introspectively adjusted behaviors reflect the ‘self-thinking’ advocated by Chinese Confucianism. Introspection is regarded as a necessary part of most psychological interventions [24]. This study found that patients’ did not possess sufficient knowledge about cancer to allow them to introspect productively during rehabilitation. The Chinese version of the Patient Learning Needs Scale (PLNS) provides a measurement tool for assessing the health education needs of discharged patients in China [25]. However it has not been used in cancer patients. This scale can be used to evaluate the learning needs of cancer patients, and individualized health education can be implemented according to the evaluation results.

#### Rebuilding self-efficacy is the key for cancer patients to return to work

In this study, the patients reported that handling work-related tasks during illness improved their confidence that helped in adaptation to return-to-work. Therefore, the patient should be familiarized with the work environment as soon as possible during or after the treatment. Medical staff can set up peer groups and invite those who return to work to meet the other patients, allowing them to get an indirect experience of adaptation to return-to-work. Medical staff should encourage patients to express their worries and persuade them to enroll in psychological counseling if required. The current study found that opting for professional psychological counseling was not common among the patients, and only one patient has consulted a professional psychologist. Addressing psychological problems of cancer patients willing to return to work may have a positive effect on the adaptation process and hospitals should work to provide access to psychological clinics to the patients to meet their mental health needs. Compared to self-management, self-efficacy emphasizes perceptions more than actual capacity or performance [18]. Self-management plan based on Bandura’s Social Cognitive Theory (SCT) use self-efficacy as a mediator to ultimately improve self-management of most patients with chronic diseases [26, 27]. Self-efficacy moderates the relationship between perceptions and activities and enhances the efficiency of self-management interventions. As a result, enhancing self-efficacy in cancer patients returning to work may be an important part of the self-management plan.

#### The adaptation plan plays an important role in cancer patients’ return to work

A work adaptation plan that considers various physical, psychological, interpersonal, and workplace specific factors is important for successful return to work [28]. Making a plan can help the patient gain clarity regarding the effort required to return to work. Medical staff should help patients to develop a suitable plan to return to work as soon as possible. Medical staff should also address specific challenges being faced by patients in non-regular employment before cancer diagnosis, as these patients may face greater challenges when trying to return to work after the treatment [29]. Cancer patients often downgrade their original career goals to maintain health. Although this can reduce the work pressure, the patients may feel dissatisfied for not being able to realize their self-worth. Hence, it is important that medical staff comprehensively evaluate each cancer patient for disease-related-, personal-, and work-related factors, compare workload capacity and work requirements to help patients maximize their professional value while maintaining health.

### Use of resource by cancer patients to return to work

The adaptation of cancer patients to return to work is primarily a process of rebuilding their lives by making most of the available resources. Personal resources including faith, resilience, beliefs, and cognition. More than half of the patients in this study regard their relationship with young children as motivating factor for returning to work so that they can provide a better life to them. Resilience enables individuals to recover from and cope with severe stress and danger [30]. This study found that resilience can help patients choose positive coping style for adapting to return-to-work. Resilience has been shown to effectively encourage return-to-work in patients with chronic pain and trauma [31]. Researchers should focus more on the role of psychological factors in the return to work of cancer patients. In this study, cancer patients with religious beliefs reported gaining positive psychological experiences such as reduced mental pain, improved sleep quality, and better interpersonal communications. Earlier studies also report that cancer patients with religious beliefs have better tolerance and higher quality of life [32, 33]. We also found that good cognition can help patients self-regulate their behavior resulting in positive outcomes related to return to work. Therefore, follow-up research can focus on developing cognitive-behavioral interventions to help patients rebuild positive awareness and promote self-regulation to adapt to return-to-work.

The external resources that are important for the return-to-work adaptation of cancer patients are family-, professional-, workplace-, and peer support. Social support has been shown to promote adaptive changes in cancer patients [34]. This is in line with the social network theory [35], which propounds that individuals get help through social relations and interpersonal communications. Cancer patients adapting to return to work require joint efforts from various groups. Family members need to establish a correct concept of recovery and help patients return to work and society as soon as possible. Studies have shown that professional support increases the quality of life in cancer patients [36]. However, most cancer patients fail to get professional help, particularly related to the mobilization of social resources matching their needs [37]. Therefore, hospitals should focus on meeting the professional needs of discharged patients to make their return to work adaptation experience less stressful. It was mentioned by a significant number of patients in this study that keeping in touch with the workplace and solving work-related tasks during sick leave increase their confidence in return-to-work. Studies have shown that workplace support is an essential factor determining cancer patients’ return to work [38].

It is important that the workplace should keep in touch with the patients during sick leave and allow them to gradually return to work. Support of workplace according to the patient’s specific physical, emotional, and personal requirements will further help cancer patients to return to work and avoid job loss.

As employers do not guarantee a return to work after illness, some patients choose to hide their illness. In this context, it may be useful to develop policies guaranteeing return to work for cancer patients. For example, in Belgium Flemish parliamentary legislation guarantees compensation during sick leave and provides a return to work protection for the patients [39] to prevent the financial distress caused by illness.

### Clinical implications

Thus, to promote return-to-work and provide a favorable adaptation experience for cancer patients, it is imperative that medical staff, vocational rehabilitation specialists, employers, and cancer patients jointly develop a return-to-work plan based on each patient’s recovery. This will help patients realize their self-worth and contribute positively to society.

### Study limitations

There are certain limitations of this study that can be addressed in future studies. This study did not look into the differences in return-to-work experiences of patients with different types and stages of cancer. The treatment of cancer varies according to the type and stage of cancer, which result in different side effects and thus different challenges during return-to-work. Also, the included patients were from a single region of China which can affect the generalization of results to other regions with different socioeconomic conditions. We have also not categorized patients according to age in this study; age can have a significant influence on the return-to-work outlook and challenges of a person. In the future, researchers can further explore the return-to-work experience of cancer patients of different ages, different types of diseases, and different stages of the disease.

## Conclusion

This study documented the return to work adaptation experience of cancer patients. The methodologies of grounded theory were used to explore the challenges faced by the patients and the internal and external coping resources used by the patients to return to work. Cancer patients returning to work face the challenge of maintaining a balance between the demands of work and health. Patients learn new healthy behaviors through introspection and learning and lay emphasis on building self-efficacy by learning from others who have returned to work after illness. Religious beliefs can help the patients attain a positive frame of mind and help return to work adaptation. Emotional support from the family, peers, and rehabilitation staff, and encouragement from superiors and colleagues from workplace are important for a positive return-to-work experience. Cancer patients returning to work also need significant adjustments to their pre-illness plans, including seeking support from colleagues and superiors at work place and scaling down their career objectives. Cancer patients use a number of internal and external resources to make the return to work possible. The findings of this study can help the professionals involved in the care of cancer patients to devise strategies to facilitate successful return-to-work of cancer patients.

## Data Availability

The datasets used and/or analysed during the current study available from the corresponding author on reasonable request.
